# Calcium, an Emerging Intracellular Messenger for the Hippo Pathway Regulation

**DOI:** 10.3389/fcell.2021.694828

**Published:** 2021-06-29

**Authors:** Yiju Wei, Wei Li

**Affiliations:** ^1^Division of Hematology and Oncology, Department of Pediatrics, Penn State College of Medicine, Hershey, PA, United States; ^2^Department of Biochemistry and Molecular Biology, Penn State College of Medicine, Hershey, PA, United States

**Keywords:** Hippo pathway, calcium signaling, actin cytoskeleton, CaAR, protein kinase C, NEDD4L, Merlin

## Abstract

The Hippo pathway is a conserved signaling network regulating organ development and tissue homeostasis. Dysfunction of this pathway may lead to various diseases, such as regeneration defect and cancer. Studies over the past decade have found various extracellular and intracellular signals that can regulate this pathway. Among them, calcium (Ca^2+^) is emerging as a potential messenger that can transduce certain signals, such as the mechanical cue, to the main signaling machinery. In this process, rearrangement of the actin cytoskeleton, such as calcium-activated actin reset (CaAR), may construct actin filaments at the cell cortex or other subcellular domains that provide a scaffold to launch Hippo pathway activators. This article will review studies demonstrating Ca^2+^-mediated Hippo pathway modulation and discuss its implication in understanding the role of actin cytoskeleton in regulating the Hippo pathway.

## Introduction

The Hippo pathway is a conserved signaling network governing organ development and tissue homeostasis. Various diseases, such as distorted tissue regeneration and cancer, have been linked to dysfunction of this pathway (Zanconato et al., 2016; Ma et al., 2019). The Hippo pathway contains a core serine/threonine kinase cascade, which, in mammals, includes Mammalian Sterile 20-like kinase 1 and 2 (MST1 and MST2) as well as their substrates Large Tumor Suppressor kinase 1 and 2 (Lats1 and Lats2, denoted as Lats1/2 hereafter). Upstream signals could induce phosphorylation and activation of Lats1/2 (denoted as the Hippo pathway activation hereafter), which in turn phosphorylate and inhibit two paralogous transcriptional coactivators, Yes-associated protein (YAP) and transcriptional co-activator with PDZ-binding motif (TAZ), by preventing their accumulation in the nucleus. Without this regulation, YAP/TAZ can accumulate in the nucleus and activate gene transcription largely through TEAD transcriptional factors.

Growth control or homeostatic signals emitted extracellularly or intracellularly can regulate YAP/TAZ through Lats1/2-dependent or -independent mechanisms. Previous studies have found that the Hippo pathway can respond multiple upstream signals, including mechanical forces, cell polarity and adhesion, soluble factors, as well as various cellular stress. The Hippo pathway regulation by these signals have been extensively reviewed recently (Ma et al., 2019). Ca^2+^ is a signaling messenger important for a variety of cellular functions. Recent studies found that Ca^2+^ can regulate YAP/TAZ in various situations and is an emerging signal for the Hippo pathway regulation. This article will review the studies supporting this notion and discuss the potential mechanism underlying regulation of the Hippo pathway by Ca^2+^.

## Ca^2+^ Signaling Activates the Hippo Pathway

**FIGURE 1:**
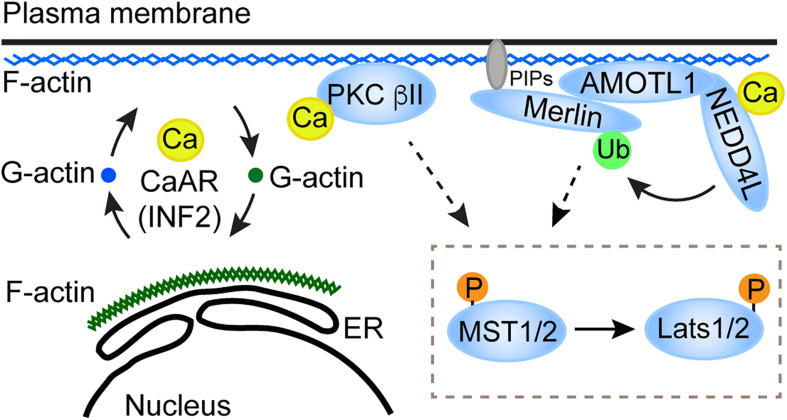
A proposed model for the role of CaAR in the Hippo pathway activation. CaAR constructs a F-actin scaffold at the cell cortex. On the one hand, the F-actin scaffold recruits and activates PKC beta II; on the other hand, the F-actin scaffold can recruit the AMOTL1-NEDD4L E3 ligase apparatus, which then activates Merlin. Both of these two signaling effectors then activate the core kinase cascade in the Hippo pathway.

In an unbiased screen of 1650 compounds, Liu et al. found that an L-type calcium channel blocker, amlodipine, is able to inhibit survival of glioblastoma cells by suppressing YAP/TAZ (Liu et al., 2019). Instead of its known function as an L-type calcium channel blocker, amlodipine can increase intracellular Ca^2+^ level by enhancing store-operated Ca^2+^ entry (SOCE) (Liu et al., 2019; Johnson et al., 2020). The elevated intracellular Ca^2+^ level inhibits YAP/TAZ by activating the core kinase cascade of the Hippo pathway. In this process, inverted formin-2 (INF2)-mediated Ca^2+^-induced actin remodeling drives accumulation of protein kinase C (PKC) beta II in an actin cytoskeletal compartment. Such translocation is critical for PKC beta II to activate Lats1/2 (Figure 1). The study suggested that Ca^2+^ is an intracellular cue that regulates the Hippo pathway. In line with this notion, knockout of two-pore channel 2 (TRC2), a Ca^2+^ channel responsible for Ca^2+^ releasing from acidic organelles (Calcraft et al., 2009), in metastatic melanoma cells increases YAP/TAZ activity (D’Amore et al., 2020). In these TRC2 knockout cells, expression of ORAI1, the plasma membrane Ca^2+^ channel responsible for SOCE, and PKC beta II was decreased. Overexpression of ORAI1 in TRC2 knockout cells reverses the effect of TRC2 depletion on YAP/TAZ target gene expression, suggesting that ORAI1 inhibition is responsible for TRC2 depletion-induced YAP/TAZ activation (D’Amore et al., 2020). In breast cancer cells, expression of secretory pathway Ca^2+^-ATPase 2 (SPCA2) can inhibit the epithelial-to-mesenchymal transition. This is through increasing cellular Ca^2+^ level and expression of E-cadherin, which then promotes YAP phosphorylation through activating Lats1/2 (Dang et al., 2019).

In addition to responding to increased Ca^2+^ level caused by directly perturbating Ca^2+^ channels, the Hippo pathway regulation by mechanical force appears to involve Ca^2+^. He et al. (2018) found that human fibrosarcoma HT1080 cells compressed by a microfluidic device can reduce RhoA activity through a Ca^2+^-dependent manner. In this process, transient receptor potential cation channel subfamily V member 4 (TRPV4) is responsible for the mechanical compression-induced Ca^2+^ influx and RhoA inhibition. Along with the reduction of RhoA activity, YAP translocates to cytoplasm from the nucleus when cells are compressed. However, the YAP translocation is suppressed when Ca^2+^ is eliminated from the compressed cells (He et al., 2018). In human adipose derived stem cells, synchronized thermal and mechanical stimulation can increase intracellular Ca^2+^ level and inhibit YAP nuclear localization (Deng et al., 2020). Inhibition of Ca^2+^ influx reduces YAP phosphorylation and increases YAP nuclear localization. In a study of the relationship among patterns of mechanical stress, bioelectric field and proliferation, Silver et al. (2020) found that mechanical stress gradients in mammary epithelial tissues of defined geometry lead more YAP/TAZ nuclear localization in cells at the tissue periphery than the center region. This phenomenon was accompanied by an increase of Ca^2+^ level in cells at this region. Interestingly, when Ca^2+^ was chelated, YAP nuclear localization was no longer limited to cells at the tissue periphery, but also occurred in the tissue center region. How Ca^2+^ determines the specific pattern of YAP nuclear localization in the epithelial tissue is unclear.

In addition to the in vitro observations, regulation of the Hippo pathway by Ca^2+^ has been recently suggested in genetic studies of the human autosomal dominant polycystic kidney disease (ADPKD) and Drosophila wing epithelium development. ADPKD is caused by mutations in *PKD1* and *PKD2* genes. Their protein products PC1 and PC2, respectively, form a plasma membrane calcium channel complex (Koulen et al., 2002; Lemos and Ehrlich, 2018). It was shown that YAP is activated in ADPKD patients and the *Pkd1*-depleted mouse (Happe et al., 2011; Cai et al., 2018). Loss of YAP/TAZ is able to suppress cystogenesis (an ADPKD-associated symptom) in the ADPKD mouse model (Cai et al., 2018). These observations suggested that the PC1/2 calcium channel complex is involved in suppressing YAP/TAZ to ensure normal kidney functions. In Drosophila wing epithelium, simultaneous deficiency in both sarcoplasmic-endoplasmic reticulum ATPase (SERCA), an endoplasmic reticulum (ER) calcium pump, and Orai leads to increased tissue growth (Suisse and Treisman, 2019). Such tissue hypergrowth is accompanied by dislocation and loss of activity of Fat, a component of the Hippo pathway (Suisse and Treisman, 2019). The observation suggested that Ca^2+^ is required for the proper activation of the Hippo pathway during the wing epithelium development.

Overall, the above studies indicated that increase of intracellular Ca^2+^ level can inhibit YAP/TAZ activity, and that Ca^2+^ may be an intracellular messenger to transduce the mechanical cue to the Hippo pathway.

## Ca^2+^ Signaling Inhibits the Hippo Pathway

In human neural stem/progenitor cells, spontaneous Ca^2+^ transients were observed at the plasma membrane when cells grow on glass coverslips (Pathak et al., 2014). The Ca^2+^ transients require Ca^2+^ influx across the plasma membrane, and its magnitude positively correlates with the substrate stiffness. Because YAP preferentially localizes in the nucleus when cells grow on a stiff surface (Dupont et al., 2011), this observation suggested a connection between the Ca^2+^ transients and YAP nuclear localization. The traction force-induced Ca^2+^ transients and YAP nuclear localization require a stretch-activated ion channel Piezo1 because knockdown of Piezo1 eliminates both events (Pathak et al., 2014). This study suggested that mechanically activated Ca^2+^ influx through Piezo1 is required for YAP nuclear localization when cells grow on a stiff surface. The response of YAP localization to Ca^2+^ influx under this cellular mechanical circumstance appears to be opposite to those described above when cells are compressed (He et al., 2018; Deng et al., 2020). Notably, the mechanical force comes from the cell cortex when cells are compressed, whereas the force is applied to the cell basal membrane when cells grow on stiff surfaces. The distinct responses in these studies indicated that YAP regulation of Ca^2+^ at the different subcellular domains may be different. Although Ca^2+^ influx occurs in both situations, different Ca^2+^ channels are involved. Ca^2+^ signals initiated by these different channels may regulate the actin cytoskeleton through distinct effectors. Furthermore, the actin cytoskeleton rearrangement at different subcellular domains may have distinct impacts on YAP activity (see more discussion below).

In a study of how cholesterol induces hepatosteatosis transition to fibrotic non-alcoholic steatohepatitis (NASH), Wang et al. (2020) found that cholesterol can upregulate TAZ by inducing its dephosphorylation and stabilization in hepatocytes. This is through activating soluble adenylyl cyclase (sAC) by internalized cholesterol and in turn activating the cAMP-PKA signaling axis. PKA then induces ER Ca^2+^ release through activating inositol triphosphate receptor (IP3R), resulting in activation of RhoA, which can then activate TAZ through inhibiting Lats1/2. Ca^2+^ signals derived from ER perturbation can also regulate the Hippo pathway in *Drosophila* wing development. Ma et al. (2020) found that a loss of function mutation of Emei, an ER Ca^2+^ regulator, can synergize with Ras^*V12*^ to induce tumor growth through inhibiting the Hippo pathway. In this process, Emei mutation reduces Ca^2+^ level in ER and subsequently activates JNK. Perturbation of another ER Ca^2+^ regulator, SERCA, indicated that reducing ER Ca^2+^ can synergize with Ras^*V12*^ to promote tumor growth. Because preventing cytosolic Ca^2+^ from importing into ER by disrupting these ER Ca^2+^ importers may cause ER Ca^2+^ imbalance, which can trigger plasma membrane Orai Ca^2+^ channel through Stim to import extracellular Ca^2+^, the Hippo pathway response in this case could be a consequence of increasing the cytosolic Ca^2+^ level. In this study, whether the cytosolic Ca^2+^ level is increased has not been directly tested. Notably, the aforementioned study reported that cells with SERCA and Orai double mutations in the wing epithelium also show an disrupted Hippo pathway (Suisse and Treisman, 2019), suggesting that SOCE induced by SERCA deficiency may not be required for suppressing the Hippo pathway. Therefore, it is possible that Ca^2+^ loss in ER and increase in the cytosolic compartment may regulate the Hippo pathway through different effectors. These studies emphasized that the Hippo pathway response to Ca^2+^ may be complex.

The complexity of the Hippo pathway regulation by Ca^2+^ was further demonstrated by a recent study through a real-time single-cell visualization of YAP subcellular localization and its target gene transcription dynamics (Franklin et al., 2020). In MCF10A human mammary epithelial cells, Franklin et al., observed a cycle of fast exportation and importation of YAP from the nucleus in response to the treatment of Ca^2+^ mobilizers, such as thapsigargin, ionomycin or ATP. Consistent with the previous observation (Liu et al., 2019), YAP exportation from the nucleus is induced by elevation of intracellular Ca^2+^ level in a Lats1/2- and PKC-dependent manner (Franklin et al., 2020). Interestingly, YAP re-enters the nucleus after the initial Ca^2+^ spike, and this nuclear re-entering can stimulate the expression of YAP target genes. Such Ca^2+^-induced YAP localization-resets suggests that YAP activity may not be simply predicted by its nuclear or cytoplasmic localization, but rely on its nucleocytoplasmic shuttling (Franklin et al., 2020). Although this notion suggested that Ca^2+^-induced YAP nuclear exportation leads to YAP activation upon its nuclear reentry, the canonical cytoplasmic YAP regulation, including tethering to 14-3-3 and ubiquitination-mediated degradation, may counteract the nuclear reentry and contribute an inhibitory effect on YAP activity. These canonical cytoplasmic inhibitory effects may be even stronger for TAZ, because TAZ appears to be less stable than YAP and its expression was markedly decreased within 30 min after Ca^2+^ influx when LN229 cells are treated by thapsigargin or ionomycin (Liu et al., 2019). Therefore, the eventual impact of Ca^2+^ on YAP/TAZ may rely on the combination of these factors. The relative contributions of these factors may vary among different cells.

Overall, the above studies indicated that the response of YAP/TAZ to Ca^2+^ may be affected by the subcellular domains where Ca^2+^ signal is initiated and the interrelations among different regulatory modules.

## Mechanism of the Hippo Pathway Regulation by Ca^2+^ and Actin Cytoskeleton

### Ca^2+^ Effectors Involved in the Hippo Pathway Regulation

It is still unclear how Ca^2+^ signaling and the core Hippo pathway components are linked together. Conventional PKC is a Ca^2+^ effector that is required for Lats1/2 activation (Liu et al., 2019; Franklin et al., 2020). The role of PKC in the Hippo pathway regulation was also reported when the pathway responds to 12-O-tetradecanoylphorbol-13-acetate or G-protein-coupled receptors perturbation (Gong et al., 2015). Whether PKC directly phosphorylates Lats1/2 or other components required for Lats1/2 activation remains to be determined. Besides PKC, an E3 ubiquitin ligase, neural precursor cell-expressed developmentally downregulated protein four like (NEDD4L) could be another Ca^2+^ effector in the Hippo pathway regulation. Recently, Wei et al., found that elevation of intracellular Ca^2+^ level or loss of matrix attachment in human glioblastoma cells and mouse Schwann cells triggers ubiquitination of Merlin (Wei et al., 2020), an essential component in the Hippo pathway for Lats1/2 activation. This process is mediated by NEDD4L and a scaffold protein, AMOTL1 (Figure 1). The ubiquitination is required for Merlin to interact and activate Lats1 in response to upstream signals, including Ca^2+^. Because Merlin-Lats1/2 interaction is important for Lats1/2 activation (Yin et al., 2013), Merlin ubiquitination appears to promote Lats1 activation through facilitating the interaction between Merlin and Lats1 (Wei et al., 2020). Currently, it is still unclear how Merlin ubiquitination can promote its interaction with Lats1. NEDD4L contains a C2 Ca^2+^-binding domain and can be activated by Ca^2+^ (Goel et al., 2015). It was suggested that the C2 domain works as an auto inhibitor of NEDD4L, and that Ca^2+^ binding to the C2 domain disrupts the auto inhibitory function (Escobedo et al., 2014). It would be interesting to determine whether the C2 domain in NEDD4L is important for its function in Merlin activation in response to Ca^2+^. Since both PKC beta II and NEDD4L have the C2 Ca^2+^-binding domain and could be directly activated by Ca^2+^, whether the two Ca^2+^-activated events (activation of PKC beta II and Merlin) are interrelated or independent needs to be resolved.

### CaAR Is Involved in the Hippo Pathway Regulation

The actin cytoskeleton appears to be a central mediator of various upstream biochemical and mechanical signals to YAP/TAZ through the core kinase cascade-dependent and -independent manners. How the cytoskeleton executes this role is unclear (Halder et al., 2012; Sun and Irvine, 2016; Fulford et al., 2018; Seo and Kim, 2018). Because of the apparent correlation between filamentous actin (F-actin) in stress fibers and YAP/TAZ activation, an inhibitory factors-sequestering model was proposed (Halder et al., 2012). This model suggested that stress fibers or other unknown F-actin networks associating with cell spreading serve as compartments sequestering YAP/TAZ inhibitory factors. Consistent with this model, disruption of stress fibers leads to Lats1/2 activation and YAP/TAZ inactivation (Halder et al., 2012; Sun and Irvine, 2016; Seo and Kim, 2018). During this process, the Merlin-Lats1 interaction (Yin et al., 2013) and the angiomotin-YAP interaction (Mana-Capelli et al., 2014) are enhanced. While Merlin and angiomotin provided clues to probe the regulation of Lats1/2-YAP/TAZ by F-actin, the detailed mechanism to reconcile their roles with the inhibitory factors-sequestering model remains elusive (Seo and Kim, 2018). Genetic studies in Drosophila found that the spectrin cytoskeleton at the cell cortex is required for the Hippo pathway activation (Deng et al., 2015; Fletcher et al., 2015; Wong et al., 2015). These studies suggested that certain cytoskeleton at the cell cortex is involved in activating the Hippo signaling. Notably, spectrin and actin are major proteins composing an integrated cytoskeleton network at the cell cortex (Holley and Ashmore, 1990). Therefore, it is plausible that loss of spectrin may interfere with the Hippo pathway activation through disrupting this cytoskeleton network.

Ca^2+^ can induce a characteristic actin rearrangement (Shao et al., 2015; Wales et al., 2016). In this process, the apical cortex actin transiently relocates to the perinuclear rim as well as ER, and rapidly reverts to the cortical distribution. Such actin remodeling could complete within 2 min of Ca^2+^ being increased and was called calcium-mediated actin reset (CaAR) (Shao et al., 2015; Wales et al., 2016). A formin family protein, INF2, is important for the actin polymerization during CaAR (Shao et al., 2015; Wales et al., 2016). In INF2-depleted glioblastoma cells, CaAR is disrupted and Ca^2+^-induced phosphorylation of Lats1 and YAP is compromised, suggesting that CaAR is important for the Hippo pathway activation by Ca^2+^ (Liu et al., 2019). It was reported that PKC beta II is activated by binding to F-actin through its actin binding motif (Blobe et al., 1996). Therefore, Ca^2+^ might induce the association of PKC beta II with F-actin during CaAR. Consistent with this notion, subcellular fractionation revealed an increased amount of PKC beta II in the Triton X-100 insoluble actin cytoskeletal compartment in response to the increase of Ca^2+^ level in cells. Knockdown of INF2 eliminates PKC beta II from this actin compartment, suggesting that INF2-mediated actin assembly is required for such PKC beta II translocation (Liu et al., 2019). Collectively, these results suggested that INF2-mediated CaAR induces PKC beta II translocation to certain actin compartments and to activate the Hippo pathway. Future studies need to determine whether there is a connection between the actin compartment involved in CaAR-induced PKC activation and the spectrin-involved cytoskeleton network at the cell cortex. Notably, it was reported that thapsigargin- or ionomycin-induced Ca^2+^ influx can promote RhoA activation and increase actin stress fiber formation in human umbilical vein endothelial cells (HUVEC) (Masiero et al., 1999). Such phenomenon relies on specific matrix where the cells grow, because it occurs in cells plated on type IV collagen, but not on type I collagen. Experimental setting may explain the distinct actin filament rearrangements in response to Ca^2+^ in CaAR and this case. Alternatively, the difference may reflect the cellular response to Ca^2+^ at acute and adapted stages, respectively, because CaAR occurs within a couple of minutes (Shao et al., 2015; Wales et al., 2016), whereas stress fiber forms at 30–90 min after the treatments (Masiero et al., 1999). As the actin filaments change their subcellular locations and properties at different stages after Ca^2+^ influx, YAP/TAZ may change their behaviors in a dynamic manner. The functional consequence of YAP/TAZ in transcriptional control may therefore be an accumulative result of such dynamic changes of YAP/TAZ activities.

## Conclusion and Outlook

Multiple studies in various circumstances have demonstrated that changes of intracellular Ca^2+^ level can regulate the Hippo pathway (Table 1). These findings suggested that Ca^2+^ could be an intracellular messenger for the Hippo pathway in responding to upstream signals, such as mechanical forces and soluble factors. Intriguingly, the Hippo pathway responses to Ca^2+^ appear to be variable when examined in different experimental settings. The distinct responses suggested that the Hippo pathway regulation by Ca^2+^ is complex, and that the Hippo pathway responses may be determined by the stages after the initial Ca^2+^ influx as well as the subcellular domains where Ca^2+^ influx occurs. Therefore, it is important for the future study to examine these temporal and spatial factors when assessing the Hippo pathway responses as well as Ca^2+^ effectors involved in the Hippo pathway regulation. An emerging principle of Ca^2+^ regulation of the Hippo pathway is the involvement of actin cytoskeleton rearrangement, such as INF2-mediated CaAR and RhoA-mediated stress fiber formation. The transition among these actin filaments may modulate the effectors, such as PKC and NEDD4L, which are responsible for the Hippo pathway regulation (Figure 1). Further dissecting the involved actin cytoskeleton rearrangements as well as connections between the effectors and core Hippo pathway components would help us to understand how actin cytoskeleton reorganization and related upstream signals regulate the Hippo pathway.

**TABLE 1:** Ca^2+^ signaling regulates the Hippo pathway under various circumstances.

Source of the Ca^2+^ signal	Direction of Ca^2+^ change	Effect on YAP/TAZ	Stimulator	Signaling mediator	Biological model	References
ORAI1 activation	Increase	Inhibition	Amlodipine	PKC beta II	Human glioblastoma cells	Liu et al., 2019
SERCA inhibition	Increase	Inhibition	Thapsigargin	PKC beta II	Human glioblastoma cells	Liu et al., 2019
		Activation			Human mammary epithelial cells	Franklin et al., 2020
Ionophore	Increase	Inhibition	Ionomycin	PKC beta II	Human glioblastoma cells	Liu et al., 2019
		Activation			Human mammary epithelial cells	Franklin et al., 2020
Not examined	Increase	Activation	ATP	Unknown	Human mammary epithelial cells	Franklin et al., 2020
TRC2 knockout	unknown	Activation	N/A	ORAI1 Inhibition	Human melanoma cells	D’Amore et al., 2020
SPCA2 expression	Increase	Inhibition	N/A	E-cadherin activation	Human breast cancer cells	Dang et al., 2019
TRPV4 activation	Increase	Inhibition	Mechanical compression	RhoA inhibition	Human fibrosarcoma cells	He et al., 2018
Unknown	Increase	Inhibition	Thermal and mechanical stimulation	Unknown	human adipose derived stem cells	Deng et al., 2020
N/A	Decrease	Activation	Ca^2+^ chelator	Unknown	Human mammary epithelial	Silver et al., 2020
PC1/2 mutation	Not examined	Activation	N/A	Unknown	Mouse or human kidney	Happe et al., 2011; Cai et al., 2018
SERCA and Orai mutation	Not examined	Activation	N/A	Fat inactivation	Drosophila wing epithelium	Suisse and Treisman, 2019
Piezo1	Increase	Activation	Traction forces	Unknown	Human neural stem/progenitor cells	Pathak et al., 2014
IP3Rs	Increase	Activation	Cholesterol	RhoA activation	Mouse and human hepatocytes	Wang et al., 2020
Emei or SERCA mutation	ER Ca^2+^ decrease	Activation	N/A	JNK activation	Drosophila wing epithelium	Ma et al., 2020

## Author Contributions

YW and WL wrote the manuscript and approved the submitted version. Both authors contributed to the article and approved the submitted version.

## Conflict of Interest

The authors declare that the research was conducted in the absence of any commercial or financial relationships that could be construed as a potential conflict of interest.
